# Human rabies surveillance and control in China, 2005–2012

**DOI:** 10.1186/1471-2334-14-212

**Published:** 2014-04-18

**Authors:** Miao Song, Qing Tang, Simon Rayner, Xiao-Yan Tao, Hao Li, Zhen-Yang Guo, Xin-Xin Shen, Wen-Tao Jiao, Wei Fang, Jun Wang, Guo-Dong Liang

**Affiliations:** 1 State Key Laboratory for Infectious Disease Prevention and Control, National Institute for Viral Disease Control and Prevention, Chinese Center for Disease Control and Prevention, Beijing, People’s Republic of China; 2 Key Laboratory of Agricultural and Environmental Microbiology, Wuhan Institute of Virology, Chinese Academy of Sciences, Wuhan, Hubei, People’s Republic of China; 3 Liupanshui Vocational and Technical College, Liupanshui, Guizhou, People’s Republic of China

**Keywords:** Rabies, Surveillance, Prevention and control, Policies

## Abstract

**Background:**

Rabies reemerged in China during the 1990s with a gradual increase in the number and geographical dispersion of cases. As a consequence, a national surveillance program was introduced in 2005 to investigate the outbreak in terms of vaccination coverage, PEP treatment, and geographical and social composition.

**Methods:**

The surveillance program was coordinated at the national level by the Chinese Center for Disease Control (CCDC) with data collected by regional health centres and provincial CCDCs, and from other official sources. Various statistical and multivariate analysis techniques were then used to evaluate the role and significance of implemented policies and strategies related to rabies prevention and control over this period.

**Results:**

From 2005–2012, 19,221 cases were reported across 30 provinces, but these primarily occurred in rural areas of southern and eastern China, and were predominantly associated with farmers, students and preschool children. In particular, detailed analysis of fatalities reported from 2010 to 2011 shows they were associated with very low rates of post exposure treatment compared to the cases with standard PEP. Nevertheless, regulation of post-exposure prophylaxis quality, together with improved management and vaccination of domesticated animals, has improved prevention and control of rabies.

**Conclusions:**

The various control policies implemented by the government has played a key role in reducing rabies incidences in China. However, level of PEP treatment varies according to sex, age, degree and site of exposure, as well as the source of infection. Regulation of PEP quality together with improved management and vaccination of domesticated animals have also helped to improve prevention and control of rabies.

## Background

The government began recording human rabies cases in China in 1950 although, initially, only the number of cases at the national level was reported. Nevertheless, the data reveals that rabies has been responsible for hundreds to thousands of fatalities in most years since recordkeeping began. Towards the end of the last century, rabies reemerged in China and became the leading cause of fatalities among the 37 notifiable infectious diseases recognized by the Ministry of Health (MOH). As a consequence, a national rabies surveillance program was implemented in 2005 in provinces and cities reporting the highest case rates [1]. The goals of this program included identifying factors driving the epidemic, the populations most at risk and the effectiveness of current control measures. Based on the initial findings, the surveillance program was subsequently expanded to encompass all epidemic regions in the country. At the same time, the national government, together with the health, agriculture, public security and drug supervision sectors, used these findings in the implementation of a number of preliminary control measures. As a consequence, from 2008, the number of fatalities case number began to fall and by 2012 the number of fatalities had dropped by 57% compared to 2007. In this report we perform a detailed examination of the data collected over the past seven years and discuss the progress in prevention and control of rabies in China.

## Methods

### Rabies surveillance in China

**Figure 1:**
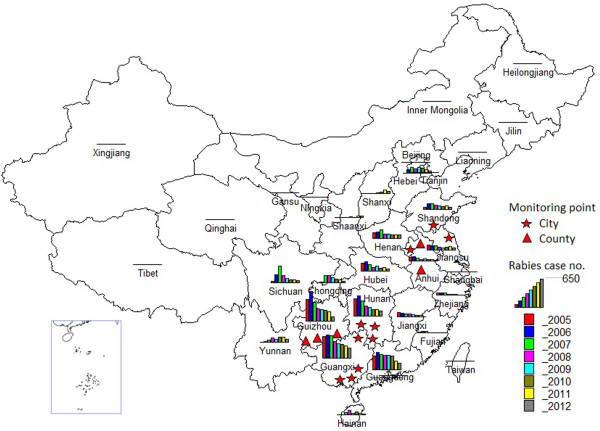
Rabies surveillance regions and geographical distribution of cases in for each province in China from 2005 to 2012.

The China rabies surveillance program was introduced in 2005 in 15 high-incidence cities and counties from six provinces reporting continuously high incidences of rabies (Figure 1). The surveillance region was expanded as the number of reported cases began to increase and spread into new areas. Currently, all regions reporting rabies have been incorporated into the surveillance system. In the event of a rabies case, a report must be submitted within 24 hours to the surveillance program via the internet. This requires completion of a “*Rabies Case investigation Questionnaire*” to supply the initial information related to the case, such as animal exposure history and PEP treatment. If possible, information regarding animal host will also be collected and followed up with laboratory testing if a sample can be collected.

To investigate the immunization coverage in domestic dogs and cats, both an ecological survey (collection of population and vaccination data from local agricultural departments) and a household survey (collection of relevant data from local households by CDC staff) were performed at 15 surveillance sites between 2005 and 2011. Data on 19,208,488 dogs and 4,067,754 cats were collected by ecological survey, and 98,392 dogs and 97,697 cats were collected by household survey.

### Government policies for rabies prevention and control

The implemented rabies prevention and control policies required cooperation between various ministries and bodies at the national level and in 2003, the MOH, the Ministry of Public Security, the Ministry of Agriculture (MOA) and the State Food and Drug Administration (SFDA) each published reports and recommendations for rabies prevention and control with a view to establishing a consolidated national policy. In particular: (i) the SFDA implemented a program for the batch release of rabies vaccine for better tracking of vaccine production and quality control and revised minimum acceptable standards for quality of manufactured human vaccine in China, (ii) the MOH examined the plausibility of integrating PEP costs into the new rural cooperative medical service to improve PEP treatment in rural areas [2], (iii) the closer integration of dog management and vaccination efforts by the MOA and Ministry of Public Security.

### Statistical analysis

Various statistical methods were used to investigate the data collected from surveillance program and from other official sources. In particular, significant differences or correlations in the temporal, spatial and population distribution were considered using the chi-squared test. Logistic regression was used to identify relationships between PEP and degree of exposure and other influencing factors. The Pearson chi-square test and Exact test were applied to identify key differences in PEP between population or social factors. We also used the chi-squared test to consider whether the introduction of the rural cooperative medical care program had produced significant changes in the percentage of people seeking PEP after a potential rabies exposure event.

### Ethics statement

The program for collection of human brain samples was approved by the Ethical Committee of the National Institute of Viral Disease Control and Prevention, China CDC, which is the national referral center for rabies diagnosis. Due to their medical condition, subjects were unable to provide consent once a rabies infection was suspected and so written informed consent was obtained in all cases from their relatives after death.

## Results

### Epidemiology situation and analysis

**Figure 2:**
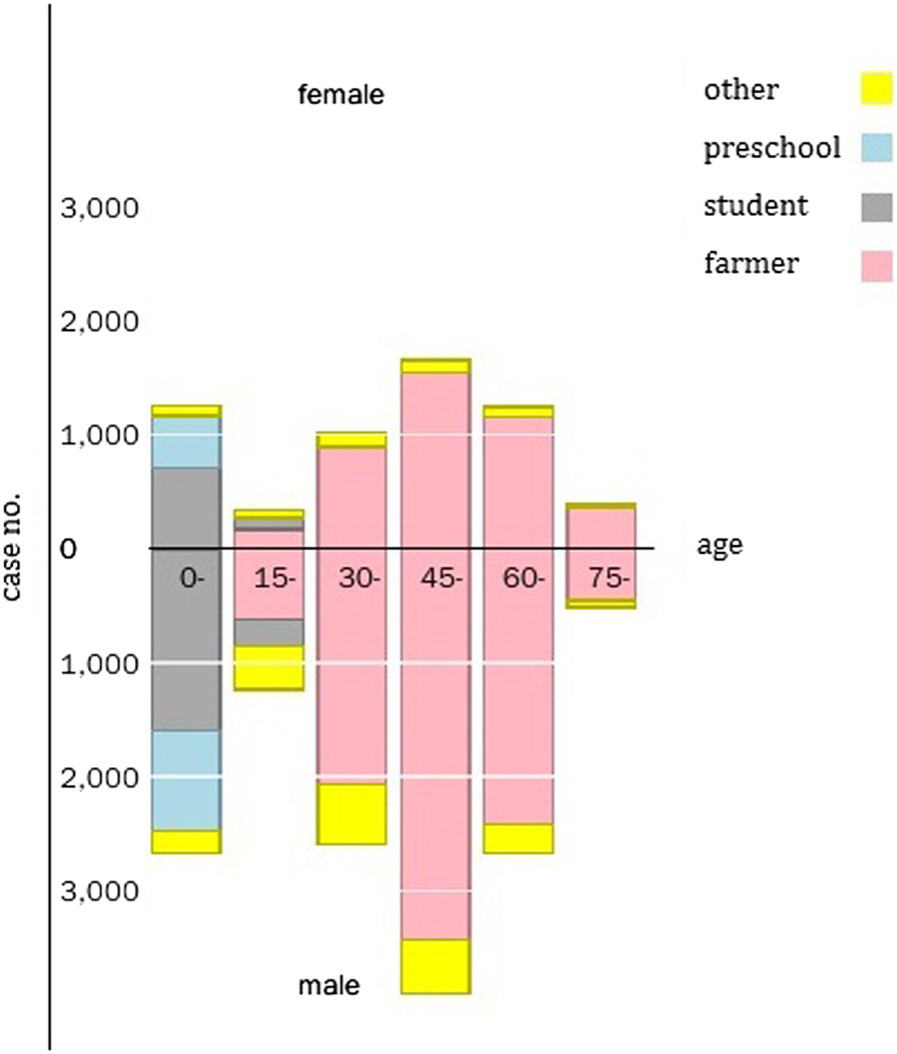
Distribution of Rabies according to age (x axis) gender (y axis) and occupation in China from 2005 to 2012.

19,221 rabies cases in humans and animals were reported in 30 provinces in China from 2005–2012 and Tibet is now the only region in China that has no reported human rabies cases. The human cases were mainly reported in the southern and eastern regions with Guangxi, Guizhou, Guangdong, Hunan and Sichuan accounting for 58.0% of the total number of cases (see Figure 1). Almost all cases occurred in rural areas (93.3%) rather than urban areas (6.7%) and were more common in males than females (2.3:1). The range of ages was from 2 months to 99 years old (median 23.5 years) with the majority of cases occurring between the age ranges of 0–14 year (s) (19.7%) and 30–74 years (81.8%). Students represent children from the age of 4 to 16 who are attending classes outside their home. From an occupational perspective, most cases occurred in farmers (67.1%), followed by students (13.3%), (typically from encountering dogs to and from school) and preschool children (6.8%) (see Figure 2). Cases were reported throughout the year, but more cases were reported in summer and autumn, especially in August, September and October (31.8%).

**Figure 3:**
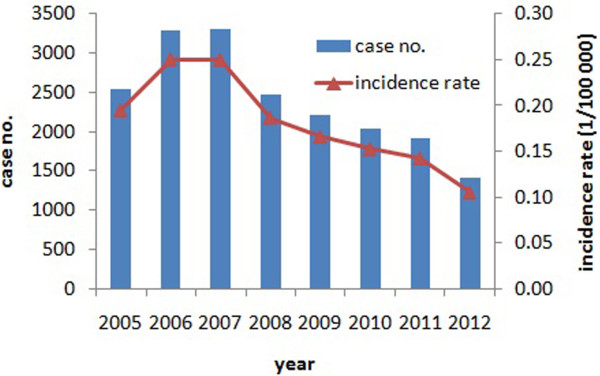
Distribution of Rabies according to age (x axis) gender (y axis) and occupation in China from 2005 to 2012.

The number of human rabies fatalities reported at the national level increased in 2005 and 2006, peaked in 2007 (3302 cases) and has subsequently declined every year (Figure 3). This trend has also been reflected in many provinces over the past 7 years, with 17 provinces in China showing a gradual decline in fatalities, most notably Guangxi, Guizhou, Guangdong, Hunan and Sichuan which were all high incidence provinces (Figure 1). These 5 provinces were responsible for 63.9% of human cases, highlighting the importance of controlling the outbreaks in these high incidence regions. However, the decrease of cases in these regions has been accompanied by reports of increasing fatalities in some western and northern regions, such as Yunnan, Shanxi, Shaanxi, Inner Mongolia and Beijing, which previously had low incidences of rabies. Most recently, in 2011–2012, rabies cases have been reported in Liaoning, Ningxia and Qinghai which have been incident free for many years (Figure 1).

Although there have been no fatalities in Tibet, in 2012, one patient was bitten by two dogs and received PEP treatment, and the brain tissue samples of the two dogs revealed the presence of rabies infection. In the same year, suspected rabies cases occurred in a dairy farm in Inner Mongolia in 2 cows that were bitten by dogs that were subsequently confirmed as infected with rabies. Finally, the first case of an imported human fatality occurred in Beijing in an Israeli national who was injured by monkeys in India and manifested the early symptoms of rabies after arriving in Beijing. Clinical diagnosis was confirmed after hospitalization and the patient died one week after the onset of the initial symptoms. The specimens of cerebrospinal fluid and saliva of the Israeli patient were sent to the laboratory of Institute for Viral Disease Control and Prevention of China CDC and tested positive for rabies.

### Human rabies cases survey and analysis

To understand the causative factors for fatalities, a retrospective survey was performed on human rabies cases in 2005–2006 [3] and, based on the results, a second survey was performed on 1302 rabies cases reported between 2010 and 2011 (Table 1). However, because of the difficulty of sample collection (most families are unwilling to permit the body of a deceased relative to undergo the necessary procedure for removing a brain sample) most reported human rabies cases are clinically diagnosed without laboratory verification, So, this survey is primarily based on epidemiological and PEP treatment data of suspected human cases between 2010 and 2011.

**Table 1:** PEP overall statistics for rabies deaths and exposed population

**Category**	**Rabies deaths**	**Exposed population**
Total number of cases	1303	1377941
By host (%)		
Dog	93.74	83.91
Cat	4.57	11.38
Exposure category (%)		
Category II	24.10	48.48
Category III	75.90	39.02
Wound treatment rate (%)		
Self treatment	27.62	16.27
Outpatient treatment	9.08	77.11
Vaccine inoculation rate (%)		
Partial inoculation	9.64	95.69
Complete inoculation treatment (5 dose)	21.90	85.34
Antibody injection rate (%)		
Injection rate for category III cases	2.96	31.24

The results showed that these human rabies cases were primarily a consequence of injures received from dogs (93.7%) and cats (4.6%) (Table 1), with the remainder associated with other domestic animals and wildlife including horses, pigs, rats and squirrels. Exposure occurred in 87.6% of cases through bites and 9.4% by scratches. Six cases were caused by the consumption or processing of dog meat. The degree of injury is classified from category I to category III, with category I the least severe (e.g., animal licking intact skin), to category II (e.g. minor scratch with or without bleeding), and category III the most severe (e.g., puncture wounds or lacerations). 75.9% of reported fatalities were category III exposure. Most of the cases with category III exposure did not receive preventive treatment according to the WHO recommendations for PEP, with few subjects receiving PEP, low rates of wound treatment (9.08%), vaccination (<10%) and immunoglobulin injection (<3%). For the category II cases, only 2 out of 267 cases were vaccinated: one patient was vaccinated on the 16th day post exposure and died on the 20th day. Also, comparison of the cases with category II and III exposures revealed there were significantly more fatalities associated with category III exposure (Pearson chi-square test and exact test, *P* = 0.0118).

### PEP treatment in suspected rabies cases

To investigate whether certain factors were associated with PEP treatment in suspected rabies cases, we performed a logistic regression with PEP as the dependent variable and sex, age, occupation, exposure category and site, and animal type (responsible for attack) as the predictors (Table 2). The results showed that sex and exposure category were the primary factors influencing the choice of PEP. A similar analysis of vaccination data indicated age, exposure category and site and type of animal were the primary determining factors, and age, exposure site and animal source were the determining factors for immunoglobulin injection (Table 3).

**Table 2:** Rabies deaths and exposed population according to age, population and gender

**Category**	**Wound treatment**		**Vaccine inoculation**		**Antibody injection**	
	**Total**	**Rate (%)**	**Total**	**Rate (%)**	**Total**	**Rate (%)**
Sex						
Male	888	31.76	867	9.11	866	1.85
Female	341	40.47	334	11.08	332	2.71
Age						
0-	218	33.03	218	18.35	218	7.34
15-	99	26.26	95	8.42	97	0
30-	199	36.18	193	6.74	196	0.51
45-	355	33.52	344	8.72	341	0.88
60-	285	38.95	282	8.51	276	1.45
75-	68	25.00	65	0	66	0
Occupation						
Farmer	884	35.18	858	7.58	861	0.93
Student	162	32.72	160	16.25	162	5.56
Preschool	79	34.18	79	21.52	77	9.09
Other	98	30.61	98	8.16	91	0
Exposure category						
Category II	267	29.59	250	6.00	253	0.40
Category III	841	38.53	811	11.96	812	2.96
Location of exposure						
Head, face, neck	128	57.03	125	46.40	127	16.54
Trunk	23	26.09	22	0	23	0
Upper limb	658	32.07	633	6.32	629	0.32
Lower limb	358	33.24	332	4.52	343	0.29
Wounding animal						
Dog	1148	34.93	1105	10.41	1100	0
Cat	55	29.09	54	1.85	55	0
Other	10	20.00	18	0	19	0
Animal origin						
Household pet	543	34.81	527	5.31	530	0.38
Neighbor’s pet	246	39.43	236	12.71	240	2.92
Roaming animal	331	32.63	319	14.42	320	4.06
Wild animal	5	20.00	2	0	3	0
Other	56	23.21	57	10.53	59	3.39

**Table 3:** Details of logistic regression analysis used to determine influential factors associated with rabies fatalities

**Y**	**X**	**β**	**S.E.**	**Wald χ**^**2**^	**P**	**OR (95% CI)**
Wound treatment	Sex	−0.38	0.142	7.134	0.008	0.684 (0.518-0.904)
	Exposure category	0.293	0.075	15.214	<0.001	1.341 (1.157-1.554)
	Overall Model Evaluation: Likelihood Ratio Test, Chi-squared 29.7357, P = 0.0001					
Vaccine inoculation	Age	0.18	0.086	4.32	0.038	1.197 (1.010-1.418)
	Exposure category	−0.643	0.322	3.994	0.046	0.526 (0.280-0.988)
	Exposure position	1.003	0.111	81.075	<0.001	2.727 (2.192-3.393)
	Animal source	−0.26	0.109	5.667	0.017	0.771 (0.623-0.955)
	Overall Model Evaluation: Likelihood Ratio Test, Chi-squared 146.3388, P < 0.0001					
Antibody injection	Age	0.503	0.216	5.401	0.02	1.653 (1.082-2.525)
	Exposure position	1.759	0.386	20.768	<0.001	5.806 (2.725-12.371)
	Animal source	−0.649	0.282	5.293	0.021	0.522 (0.300-0.908)
	Overall Model Evaluation: Likelihood Ratio Test, Chi-squared 95.9648, P < 0.0001					

Analysis of vaccination rates also revealed a number of differences in the composition of cases. Specifically, Pearson chi-squared tests and exact tests revealed that vaccination was more likely to be associated with: 1. cases occurring in children under the age of 15 compared to other groups (*P* < 0.0001). 2. category III exposure rather than category II cases (*P* = 0.0116), 3. head and neck wounds over other locations. (*P* < 0.0001) 4. cases associated with neighbor’s dogs compared to cases associated with family dogs (*P* = 0.0004), 5. cases associated with roaming dogs more than domesticated dogs (*P* = 0.0005).

Similarly, immunoglobulin injections were more likely to be associated with 1. cases occurring in children under 15 years old (*P* < 0.0001) 2. head and neck wounds over other locations (*P* < 0.0001), 3. cases associated with neighbor’s dogs compared to cases associated with family dogs (*P* = 0.0052), 4. cases associated with roaming dogs more than domesticated dogs (*P* = 0.0020).

A second PEP treatment dataset was also collected from 45 medical clinics at 15 surveillance sites that comprised 1,377,941 patients who were treated for general animal injuries (Table 4). Most of the injuries were associated with dogs (83.9%), with the remainder mainly associated with cats (11.4%). There were two notable differences with the identified human rabies cases. Firstly, most of the injured patients were category II exposure (48.5%), secondly, most of these patients (95.7%) received vaccination and wound treatment (92.1%), and 31.2% of the patients with category III exposure received an immunoglobulin injection.

**Table 4:** Injury statistics by region and host as reported from PEP clinics from 2005 to 2011

**Province**	**Number of cases**	Injury by dog (%)*	Injury by cat (%)*
Hunan	347175	86.52	6.76
Guangxi	122994	65.68	19.71
Guizhou	33643	89.89	2.15
Anhui	57891	82.69	14.38
Shandong	424542	91.24	7.37
Jiangsu	210674	70.86	24.87
Total	1196919	83.20	11.73

*Percentage of injured individuals receiving the indicated treatment.

### Surveillance of animal host for human rabies

**Figure 4:**
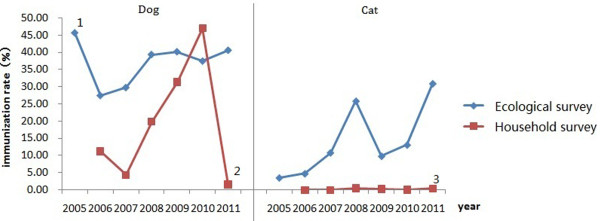
**Dog and cat immunization rates in selected surveillance regions in China from 2005 to 2012.** Notes. ^1^In 2005, only Guizhou and Guangxi provinces had available data. ^2^In 2011, only household survey data was available for dog immunization in Dushan county in Guizhou province. ^3^In 2011, only household survey data was available for cat immunization in Dushan county in Guizhou province.

Despite the evident role of dogs and cats in transmitting rabies to humans, in the absence of registration of domestic animals in China, little is known about the density and vaccination coverage of these hosts. In an attempt to estimate these quantities, population and corresponding vaccination of domestic animals were surveyed at 15 sites through consultation with local husbandry departments (ecological survey) and by household questionnaires (household survey). Based on results from both surveillance methods, the estimated dog density was 7.0/100 persons, with an immunization coverage of 36.4%; the estimated cat density was 1.8/100 persons with an immunization coverage of 15.6%. The estimates of immunization rates of dogs and cats obtained by the ecological survey were higher that estimates obtained by household survey (Z-test, *P* < 0.0001) (Figure 4).

There have also been reports of rabies cases in wildlife and subsequent human cases as a consequence of exposure to infected animals. These reports originated in Zhejiang, Anhui and Jiangxi provinces and case numbers have increased in recent years, especially human rabies cases caused by ferret badgers [4-7].

### Laboratory testing of collected isolates

**Figure 5:**
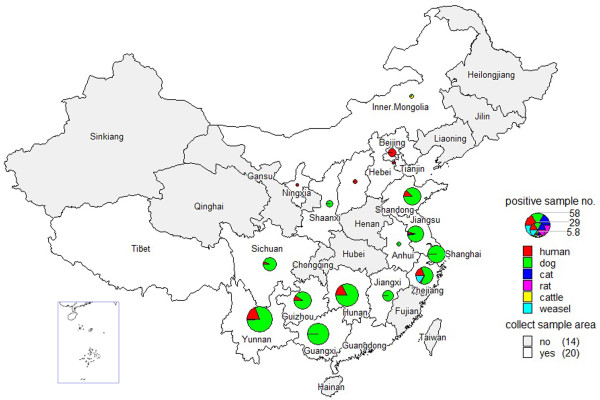
Geographical distribution of positive samples by host collected in China from 2005 to 2012.

From 2005 to 2012, 9564 samples were collected from animals that were suspected to be infected with rabies from their association with potential human rabies cases. Additionally, 140 patient samples were collected where possible to further verify diagnosis. The scope of this phase of the surveillance was gradually expanded and by 2012 samples were being collected from almost all regions reporting human rabies cases. The samples were tested using standard DFA and RT-PCR procedures [8], and a total of 364 positive samples from 18 provinces were identified. Sequences from these samples were used in a series of epidemiological studies to investigate various aspects of the current epidemic and are summarized elsewhere [8-11]. These results highlighted the emergence of a new lineage in the current epidemic that originated from within China which rapidly displaced other lineages and was almost exclusively responsible for new cases at low incidence or previously incident free regions (Figure 5). Furthermore, consideration of host species showed that dogs were the main source for human rabies viral infection, but some wildlife, such as ferret badger, have also become important reservoirs and are responsible for many reported rabies infections in livestock and other wildlife [12,13].

### Rabies prevention and control since surveillance

As discussed above, analysis of human case data showed that lack of PEP or use of non-standard PEPs is a key factor in determining the outcome of human rabies cases. The primary reasons for failing to obtain PEP is due to limited awareness of rabies and poverty levels in rural areas [14].

In an attempt to improve rabies awareness, there has been widespread introduction of education programs in rural areas. At the same time, training for diagnosis, PEP use and patient care has been implemented for medical professionals providing clinical services in local CDCs and hospitals throughout the country. To improve PEP coverage, after estimating the required annual national expenditure, in 2009 the MOH recommended integrating the costs of rabies PEP into a “new rural cooperative medical service” reimbursement program (Table 5). This has reduced the economical burden on exposed individuals and improved the PEP coverage in rural areas.

**Table 5:** Summarization of coverage and expenditure of the new rural cooperative medical (NRCM) program in China from 2005 to 2011

**Index**	**2005**	**2006**	**2007**	**2008**	**2009**	**2010**	**2011**
Number of regions incorporating NRCM program	678	1451	2451	2729	2716	2678	2637
Number of people enrolled in NRCM (×10^7^)	1.79	4.1	7.26	8.15	8.33	8.36	8.32
Percentage of target population enrolled in NRCM (%)	75.7	80.7	86.2	91.5	94.2	96.0	97.5
Financing per person (Yuan)	42.1	52.1	58.9	96.3	113.4	156.6	246.2
Total expenditure per year (hundred million Yuan)	61.8	155.8	346.6	662.3	922.9	1187.8	1710.2
Compensation benefits/10^6^ persons/year	1.22	2.72	4.53	5.85	7.59	10.87	13.15

Another important development is the observed improvement of quality and consistency of rabies vaccines manufactured in China from 2005 to 2012. In the previous major epidemic that occurred in the 1980s, only the Essen regimen was available. Since then, cell culture rabies vaccines have replaced the nerve tissue rabies vaccines, and the Zagreb regimen was approved and introduced in China in 2010. All the human rabies vaccines produced in China are purified cell culture vaccine without adjuvant. In 2010, the SFDA (State Food and Drug Administration) of China revised the China pharmacopoeia, providing officials standards (such as purity, dosage, precautions and storage) for recognized drugs, and further improved the human rabies vaccine quality requirements [15]. As a consequence of these efforts, China is now one of the largest producers and consumers of human rabies vaccine. Thus, more choices are now available for rabies vaccine administration.

## Discussion

The rabies surveillance program is now well established in China, and provides a comprehensive overview of the rabies situation throughout the country. At the same time, it is possible to use the data to review the situation within a specific region at the provincial, county or even within a specific group of villages. With this information it is possible to identify hotspots that may be associated with a local outbreak and provide the necessary control measures to prevent further spread.

Analysis of the collected data allowed the identification of rabies hotspots so they could be prioritized for control measures. Furthermore, statistical analysis revealed a number of trends that were facilitating the spread of the virus as well as increasing fatality rates. Based on these findings the government was able to implement strategies that could effectively curb further increases in fatality rates. One key factor was the identification of “at risk” populations, raising their awareness of the disease, informing them of risks from infectious animals as well as how to recognize the symptoms, and the importance of seeking PEP treatment in the event of potential exposure. Secondly, the introduction of the new rural cooperative medical subsidies for PEP costs made it easier for people in low income areas to seek treatment. Finally, introductory efforts for dog management and free immunization in high-incidence areas have highlighted the effectiveness of this approach for controlling the spread of rabies.

Although the annual number of rabies cases in China has been decreasing since 2008, this has been achieved by controlling cases in high and medium incidence provinces. The surveillance data from reported cases reveals that rabies is gradually expanding from the southern and eastern regions to the northern and western regions of China. Although the rapid economic development in China has made it possible to implement a surveillance program and fund PEP costs, it is the changes in lifestyle that are a consequence of this development that have helped to create an environment to facilitate the spread of rabies. As personal wealth has increased, so has dog ownership [16], where they generally serve as pets in cities or as guard dogs in the countryside. Furthermore, the ownership of private vehicles has also grown together with an efficient transportation infrastructure. Thus, the host population primarily responsible for human cases has not only grown rapidly, but has also become more mobile.

The question of how to manage and immunize the rapidly-increasing domestic dogs population has developed into an important social issue. Furthermore, efforts in the last decade to restore the natural environment in China has promulgated a series of laws and regulations, such as the “Wildlife Conservation Law” and the “Regulation on Terrestrial Wildlife Protection” act which has established more than 2000 nature reserves as major habitats for wild animals. Additionally, projects such as the natural forest protection project and the Grain for Green Project has prohibited hunting in these areas protecting these wildlife species [17]. Thus, while these efforts have helped to restore these natural environments, it has facilitated the rapid recovery of wildlife populations in these areas, which serve as effective reservoirs for rabies. This is supported by the increase in reported rabies cases in wildlife in these regions [12].

The situation in China contrasts with that found in North America [18] and Europe [19]. In these regions, an effective vaccination program has eliminated the virus in domestic animals and a comprehensive oral vaccine bait effort has helped to significantly control the disease in wildlife. However, repeated reports of outbreaks in local wildlife populations, raccoons in particular, e.g., [20,21] highlights the continued threat from this pathogen.

Despite the importance of PEP treatment following potential exposure to rabies, in many fatal cases patients failed to obtain this treatment. Our analysis indicates that there are two reasons for this. Firstly, in the mid-1990s, rabies had been effectively brought under control in China, with only low-incidence or scattered cases and, as public awareness of the disease fell, there was little effort directed to rabies prevention and control. Even after an attack by a dog, people rarely sought medical treatment. Secondly, even after the number of cases began to increase again and people became more aware of the importance of proper treatment, the costs were prohibitively high in many low income areas. Additionally, many health departments or clinics in villages lacked the facilities for providing PEP or had a shortage of rabies vaccine. Hence, efforts to improve awareness in the population in high incident regions as well as subsidization for PEP treatment together with education of the medical personnel for storage and delivery of PEP treatment according to WHO recommendations were key to reducing rabies cases in these regions.

Continued efforts are needed to further control rabies and to prevent its reemergence in new areas. This is remains a priority with the State Council and the relevant state departments such as the Ministries of Health, Public Security and Agriculture [22]. The State Council outlined their future plan in the document *Long-term Animal Disease Prevention and Control Plan (2012–2020*), which lists the rabies as one of 16 domesticated animal diseases to be prioritized for prevented and controlled. This plan will be implemented in part via agricultural sectors and public security departments, and includes introduction of a pilot program for identifying dogs that have been immunized and by introducing further training courses on animal rabies prevention and diagnosis for the professionals in veterinary departments [23]. All these policies and efforts will be important factors for further control of rabies in China.

## Conclusions

The national surveillance program has proved valuable for understanding the causes of the current rabies epidemic in China, as well as determining the effectiveness of the various control policies. In particular, the availability of PEP treatment is key to reducing fatalities. The results from this study should also be useful to other countries or regions facing similar challenges from rabies outbreaks.

## Competing interests

The authors declare that they have no competing interests.

## Author’s information

Miao Song is an Assistant Professor at Liupanshui Vocational and Technical College in Guizhou province. She completed her Masters degree within the State Key Laboratory for Infectious Disease Prevention and Control at the Chinese Center for Disease Control in Beijing: Her research focused on the investigation of factors contributing to the spread of rabies in China, and this remains the focus of her current research interests.

## Authors’ contributions

QT conceived of and designed this study, and MS drafted the manuscript. QT and SR revised the manuscript in detail. XYT and HL collected the data, and WF & SR performed the statistical analysis. ZYG, XXS, WTJ, JW and GDL made significant contributions to this work by providing assistance and helped in the data collection, data manipulation and analysis. All authors read and approved the final manuscript.

## Pre-publication history

The pre-publication history for this paper can be accessed here:

http://www.biomedcentral.com/1471-2334/14/212/prepub
